# Investigating the Efficacy of *Saccharomyces boulardii* in Metabolic Syndrome Treatment: A Narrative Review of What Is Known So Far

**DOI:** 10.3390/ijms241512015

**Published:** 2023-07-27

**Authors:** Mariana Buranelo Egea, Josemar Gonçalves de Oliveira Filho, Ailton Cesar Lemes

**Affiliations:** 1 Goiano Federal Institute of Education, Science and Technology, Campus Rio Verde, Rio Verde 75901-970, Brazil; 2 School of Pharmaceutical Sciences, São Paulo State University (UNESP), Araraquara 18610-034, Brazil; josemar.gooliver@gmail.com; 3 Department of Biochemical Engineering, School of Chemistry, Federal University of Rio de Janeiro (UFRJ), Rio de Janeiro 21941-909, Brazil; ailtonlemes@eq.ufrj.br

**Keywords:** yeast, probiotics, health benefits, intestinal modulation, healthy diet

## Abstract

Metabolic syndrome (MetS) is characterized by complex metabolic changes involving a cluster of co-occurring conditions, such as abdominal obesity, high blood pressure, high fasting plasma glucose, high serum triglycerides, and high LDL cholesterol levels or low HDL cholesterol levels. The incidence and risk factors of MetS occurrence increase every year. It is estimated that MetS affects approximately 30% of the population of some countries. Therefore, novel strategies are being studied to reduce the negative impact of having an unbalanced diet and a lack of physical activity. One of these strategies is the administration of probiotic microorganisms, such as the yeast *Saccharomyces boulardii*, which has been associated with several beneficial health effects (including modulation of the intestinal microbiota and improvement of the inflammatory, antioxidant, antibacterial, antitumor, and anti-inflammatory profiles). Thus, the objective of this study was to review the risk factors of MetS occurrence and the beneficial effects of *S. boulardii* ingestion in the treatment of MetS. Here, we critically evaluate the treatment necessary to promote these benefits. Using the pre-established inclusion criteria, eight studies were reviewed, including five animal and three human studies. The results reported the regulation of the lipid profile, modulation of the intestinal microbiota and gene expression, and a decrease in mass gain as positive results when *S. boulardii* was administered. Although more experiments are needed to validate these results, especially using human models, there is a trend toward improvement in MetS and a reduction in its risk factors with the administration of *S. boulardii*.

## 1. Introduction

Metabolic syndrome (MetS) is a disorder caused by energy storage and consumption [1]. It is diagnosed by the co-occurrence of three of the following five medical conditions: abdominal obesity, high blood pressure, high fasting plasma glucose, and high serum triglycerides and LDL cholesterol levels [2].

The World Health Organization (WHO) estimates that there are 650 million, 1.28 billion, and 422 million adults with obesity, hypertension, and diabetes, respectively, worldwide [3,4,5]. Furthermore, hypercholesterolemia impacts 930 million people, and 790 million people are affected by high serum triglyceride levels [6,7]. Moreover, with the COVID-19 pandemic, more negative impacts on health are predicted since there was a reduction in energy expenditure as a result of a decrease in physical activity and high food intake as a result of home confinement [8]. This can further exacerbate MetS incidence and the occurrence of its risk factors.

Concern about the quality of diet has increased with the elucidation of its direct relationship with the improvement of health and, consequently, quality of life. To reduce the occurrence of MetS and its risk factors, the addition of probiotics in the human diet has been highlighted [9].

Probiotics are live microorganism that, when administered in adequate amounts, confer health benefits on the host [10], such as regulating the host’s intestinal microbiota [11], improving symptoms of chronic gastritis [12], antimicrobial activity with elimination of pathogenic bacteria [13], and antioxidant capacity [14]. The consumption of probiotics can promote an increase in the number of beneficial microorganisms in the intestinal microbiota and reduce intestinal permeability to lipopolysaccharides (LPS) produced by Gram-negative intestinal microorganisms and/or increase the production of metabolites (e.g., short-chain fatty acids) that can help to promote health. Therefore, modulating the gut microbiota through dietary interventions (e.g., probiotic intake) may be useful for the prevention and control of metabolic disorders [15].

However, rigorous evaluation of the use of probiotics is necessary since the beneficial effects are dependent on the microorganism used, duration of treatment, dose, and route of administration [16]. Previously, Tenorio-Jiménez et al. [17] in their publication reviewed a total of nine clinical studies, six of which were randomized controlled trials (RCTs) that used a dose of 10^8^ cells/mL to 1.5 × 10^11^ colony forming units (CFU)/g of probiotics with the duration of 3–12 weeks. In general, the strains used in the RCTs were Lactobacillus or Bifidobacteria which resulted in discrete effects on some clinical characteristics of MetS and in the decrease in inflammatory biomarkers.

Thus, probiotic microorganisms have been included in the daily routine of the population that seeks a healthy life, and the yeast *S. boulardii* is a probiotic that has shown potential to protect the gastrointestinal tract from inflammatory processes [18]. *S. boulardii* is a yeast recognized as safe for application in food and pharmaceuticals. It has a high tolerance to gastric juice [19], and it can regulate the intestinal microbiota, antioxidant activity [14], and antimicrobial activity against a wide range of microbial pathogens in the intestinal region [20] with several additional benefits associated with its consumption.

Considering that it is well-known that the probiotic effects are strongly strain-dependent, to the best of our knowledge, there has not been a review published where the effects of *S. boulardii* administration in patients undergoing MetS treatment were reviewed. Thus, the objective of this study was to review the beneficial effects of *S. boulardii* ingestion in the treatment of MetS and its risk factors and to critically evaluate the treatment necessary to promote at least some benefits. We used the association of terms “metabolic syndrome”, “obesity”, “hyperlipidemia”, “dyslipidemia”, “hypertension”, “high blood pressure”, “diabetes”, and “*Saccharomyces boulardii*” in the English language, in a search of the PubMed, Scopus, and Web of Science databases in October 2022. We also checked the supporting references of the selected articles. The inclusion criteria used were the following: (1) in vivo study (with animals and/or humans); (2) intervention with *S. boulardii* alone or together with food; (3) outcomes related to MetS or its risk factors (obesity, hypertension, diabetes, and dyslipidemia); and (4) publication in English.

According to the initial search strategy, one hundred fifty-six articles were retrieved, of which nine were excluded as duplicates and one hundred forty-seven as reviews or off-topic articles. Five articles were included using a secondary search (manuscripts cited in manuscripts found by the systematic search but were cited by those included by the systematic search). After the analysis of titles and abstracts and a full-text review, eight articles were included in this review.

## 2. Metabolic Syndrome and Its Risk Factors

**Figure 1:**
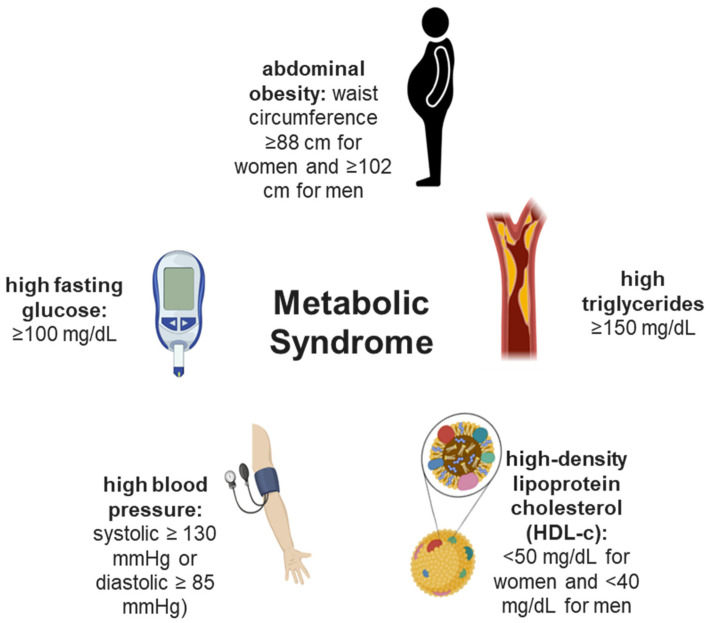
Schematic demonstrating risk factors for metabolic syndrome (MetS).

Metabolic syndrome (MetS) is characterized by the occurrence of at least three of the following five risk factors: (1) high abdominal obesity (waist circumference ≥ 88 cm for women and ≥102 cm for men), (2) elevated triglycerides (≥150 mg/dL), (3) low high-density lipoprotein cholesterol (HDL-c) (<50 mg/dL for women and <40 mg/dL for men), (4) high blood pressure (systolic ≥ 130 mmHg or diastolic ≥ 85 mmHg), and (5) elevated fasting glucose (≥100 mg/dL) (Figure 1). Each component of MetS increases the risk of cardiovascular disease, type 2 diabetes, and all-cause mortality [21].

MetS is a public health concern because it is globally highly prevalent and is associated with several health conditions [22]. The prevalence of MetS is usually associated with increasing age (>40 years) [23]. Although it is also related to dietary habits [24] and being female [25], it can also affect men of the same age [26]. To date, no drug has been approved for the treatment of MetS [27], but physical activity and dietary modification remain the best strategies [28]. Physical exercise regulates the metabolism of fat and glucose, resulting in an increase in insulin activity. In addition, it lowers blood pressure and improves blood pressure control [29]. The adoption of a balanced diet, including healthy foods—such as food low in saturated and/or trans fatty acids, salt, and sugar—and the timing of consumption, helps to restore normal metabolic profiles, contributes to weight reduction, regulates blood pressure, and controls blood glucose and lipid profile levels [30].

Obesity and overweight are the result of abnormal or excessive accumulation of fat in the human body. In 2016, it was estimated that there are 1.9 billion and 650 million people living with overweight and obesity, respectively, worldwide [3]. The occurrence of corpulence and obesity is due to an imbalance between energy consumption and expenditure. However, factors such as the environment, genetics, and infectious agents can also affect their occurrence [31].

Overweight and obesity can contribute to the manifestation of nonalcoholic fatty liver disease (NAFLD), which is defined by a liver fat content >  5% in the absence of significant alcohol consumption (characterized as 30 and 20 g/day for men and women, respectively). This arises when there is only evidence of hepatic steatosis in histology. Meanwhile, nonalcoholic steatohepatitis (NASH) occurs in the presence of steatosis and hepatic inflammation, with hepatocyte injury, and with or without perisinusoidal fibrosis [32].

Hypertriglyceridemia, which can occur in MetS, is caused by abnormal concentrations of triglycerides in the blood [33]. It affects approximately 790 million people worldwide [6]. Hypertriglyceridemia can be (i) a result of various genetic defects that lead to disordered triglyceride metabolism or (ii) an acquired cause, such as a high-fat diet, obesity, diabetes, hypothyroidism, or certain medications [34].

Hypertension, associated with visceral obesity and insulin resistance (IR), is a classic feature of MetS [35]. The number of adults with hypertension has practically doubled in 20 years (594 million to 1.28 billion from 1975 to 2021), mainly affecting low- and middle-income countries [5,36]. Adipocytes present in fat tissue can produce several biologically active peptides, including angiotensinogen (angiotensin-converting enzyme, cathepsins, and cytokines). This can subsequently lead to the onset of obesity-associated hypertension [37]. In addition, hypertension can be caused by a reduction in the body’s antioxidant system by decreasing vasodilation via the stimulation of nitric oxide (NO) [38].

High blood glucose levels, which are usually a result of insulin resistance (IR), are common in people with MetS. It is estimated that approximately 537 million adults (aged 20–79 years) live with high blood glucose levels and cause at least USD 966 billion in annual health expenditures [39]. The accumulation of lipocytes promotes the release of interleukin-6 (IL-6) and tumor necrosis factor-α (TNF-α) into circulation, subsequently stimulating the increased production of C-reactive protein (CRP). These cytokines are considered pro-inflammatory markers of obesity [40] and can alter the insulin signaling pathway, resulting in reduced glucose uptake and increased serum glucose levels [41].

The occurrence of metabolic syndromes, in addition to their deleterious effects on the health of individuals, can cause enormous damage and compromise health systems around the world. However, several strategies can be adopted to control metabolic syndrome, including diet, regular physical activity, and appropriate consumption of probiotics [42,43].

## 3. Probiotics

The term “probiotic” originates from a Greek word that translates to “for life”, meaning a substance or organism that promotes the quality of the health of the host. It was first used by Lilly and Stillwell [44] who revealed that some substances produced by microorganisms could promote the growth of other microorganisms.

Several studies have been conducted over the years to better define the concept and characteristics of probiotics. In 2001, WHO and the Food and Agriculture Organization defined probiotics as “live microorganisms which, when administered in adequate amounts, confer a health benefit on the host” [45]. Numerous microorganisms have been used as probiotics, the main ones belonging to the *Lactobacillus* and *Bifidobacterium* genera, but enterococci are also of great importance [46]. Finally, in 2014, the International Scientific Association for Probiotics and Prebiotics (ISAPP) revised the definition of probiotics as “live microorganisms that, when administered in adequate amounts, confer a beneficial effect on the health of the host” [10].

Several beneficial effects have been accepted for probiotic microorganisms, such as (a) an increase in the nutritional value of food products [47]; (b) control of and reduction in serum cholesterol [48]; (c) improvement of the immune system acting on innate and adaptive immunity (including the promotion of the development and maturation of the immune system, which increases the viability of macrophages and natural killer cells) [49]; (d) prevention of intestinal infections and suppression of antibiotic-associated diarrhea [50]; (e) reduction in symptoms of lactose intolerance [51]; (f) reduction in the risk of colon cancer [52]; and (g) improvement of gliadin digestion against celiac disease in gluten-containing probiotic foods [53]. All these effects are associated with certain strains, amounts, and periods of administration, in addition to extrinsic factors such as improved diet and physical activity [47].

Probiotics increase chemical and biological barriers in the intestinal tract, regulate the balance of the intestinal microbiota by increasing the synthesis of junction proteins between epithelial cells present in the intestine, stimulate and promote the expression and secretion of mucosal glycoproteins, increase the integrity of intestinal epithelial cells, strengthen the mechanical barrier function of the intestinal tract, and prevent translocation of intestinal bacteria and endotoxins [54].

Probiotics are used therapeutically since homeostasis of the intestinal microbiota is essential for protecting the body against diseases and infections. The broad metabolic capacity of the gut microbiome is linked to its functions. Gut microorganisms possess enzymes that allow them to use the ingested nutrients to produce a range of microbial metabolites that can affect host metabolism and health [55].

In this way, probiotics can act by correcting imbalances caused by anatomical or metabolic aggression in the intestinal mucosa [56]. However, the effectiveness of action and the reach of positive factors for the health of probiotic strains depend on the success of invasion of the barriers of the gastrointestinal tract (GIT). Thus, some points must be considered in the inclusion of probiotics, such as gastric acid, digestive enzymes, bile acids in the upper gastrointestinal tract, and colonization resistance caused by commensal bacteria in the colon [57]. These barriers can be overcome using a sufficient number of microorganisms, observation regarding the shelf life of the product, microorganisms resistant to gastric acidity, tolerance to biliary toxicity, an ability to perform transient colonization of the GIT, an ability to inhibit intestinal pathogens, and stimulation of the immune system [58].

Several bacteria and yeasts have already been identified for their probiotic power and because of their beneficial effects on the host and consequent improvement of body conditions [59,60]. Among these, *S. boulardii* stands out because of its properties and advantages which are highlighted below.

### 3.1. Saccharomyces boulardii

*S. boulardii*, the only yeast with a probiotic status, was initially isolated from the lychee fruit by Henri Boulard in 1920 [61], who observed that people who ingested lychee and mangosteen tea did not develop symptoms of cholera [14]. *S. boulardii* demonstrated high tolerance to gastric juice from simulated in vitro digestion, including tolerance to acidic pH and the effect of bile salts that can cause an antibacterial effect via the disruption of intra- and extracellular membranes [19]. Additionally, the optimal growth temperature of *S. boulardii* is 37 °C, which is the human body temperature [14].

*S. boulardii* is a nonpathogenic yeast that is clustered in a circular shape with a whitish cream color, elliptical morphology, and pseudohyphae. The yeast is thermotolerant, acid-tolerant, resistant to a simulated gastric environment and low pH (2.0 and 7.0), and is responsive to external stresses including anaerobic conditions, as well as pH and osmotic changes [62,63,64,65,66]. This makes it an important microorganism as a strategy to combat metabolic syndromes.

Administration of *S. boulardii* has been recommended mainly in the cases of diarrhea caused by *Clostridium difficile* [67] and/or in conjunction with antibiotic administration to promote recolonization of the microbiota [68] and to improve symptoms of chronic gastritis [12], among others.

*S. boulardii* seems to demonstrate a renoprotective effect, reducing albuminuria and proteinuria and attenuating the histological changes in animals with induction of diabetes (type 1 diabetes). This may be related to the reduction in hyperglycemia and may be efficient in delaying refractoriness in diabetes [69]. In addition, it has the potential to attenuate liver injury caused by the disease, reducing the concentration of carbonyl protein, increasing the activity of the antioxidant enzymes superoxide dismutase and glutathione peroxidase, and regulating the hepatic concentration of peptides responsible for the renin–angiotensin system [70].

*S. boulardii* acts as a regulator of the intestinal microbiota [14] and demonstrates antioxidant capacity that seems to occur not only in yeast but also in metabolites produced after fermentation. In addition, some proteins with different molecular weights secreted by *S. boulardii* have antimicrobial activity, being able to cleave microbial toxins, directly inhibiting the growth of the pathogen, reducing cyclic adenosine monophosphate (cAMP) production, and stimulating the activity of disaccharidases in the host mucosa [19,71,72,73]. Another feature that contributes to its antimicrobial activity is the ability of *S. boulardii* to self-aggregate and its cell surface hydrophobicity, which is essential for adhesion to the intestinal epithelium and subsequent elimination of pathogenic bacteria due to binding competition [74].

*S. boulardii* produces many bioactive metabolites (2-hydroxyisocaproic acid, gamma aminobutyric acid, shikimic acid, P-aminobenzoic acid, tyrosol, and polylactic acid) that generally have antioxidant, antibacterial, antitumor, and anti-inflammatory properties [19]. Compared with other probiotics that are normally bacteria, yeasts larger in size do not acquire antibiotic resistance [14], which may facilitate their application in the development of new food products or even help in their conservation and survival when administered in the form of a nutraceutical.

Some studies have revealed that *S. boulardii* can modulate the immune system by suppressing pro-inflammatory cascades, creating a tolerant state by acting on Toll-like receptors on dendritic cells and promoting intestinal secretory levels of IgA or trapped T cells [75]. In addition, *S. boulardii* exhibits a trophic effect on intestinal enterocytes, stimulating brush border membrane digestive enzymes and nutrient transport [76].

The ingestion of *S. boulardii* is considered safe and well-tolerated, except for a few reports of fungemia in patients with severe bowel disease and general illness [77], which appears to be related to central catheter contamination. However, this mechanism is not clearly understood [78]. Preparations with *S. boulardii* have been produced and marketed since 1950 and have been used in clinical studies since 1977; thus, this is indicative of their nonpathogenic and relatively safe nature [79].

*S. boulardii* has been increasingly explored for the development of functional foods, being incorporated into different food formats to develop products such as yogurt, cheese whey, ice cream, beer, boza, corn flakes, cashew juice, chocolate, coffee, and tea [80,81].

### 3.2. Understanding Possible Actions of Saccharomyces boulardii in Metabolic Syndrome and Its Risk Factors

Table 1 [66,82,83,84,85,86,87,88] presents the studies that met the inclusion criteria of the literature search and were reviewed to compose this narrative review article. Five of them were performed in animal models, whereas three of them were performed in human models. The dose used and the duration of the intervention varied from 10^8^–10^10^ colony forming units (CFU)/g and from 2 to 12 weeks, respectively.

**Figure 2:**
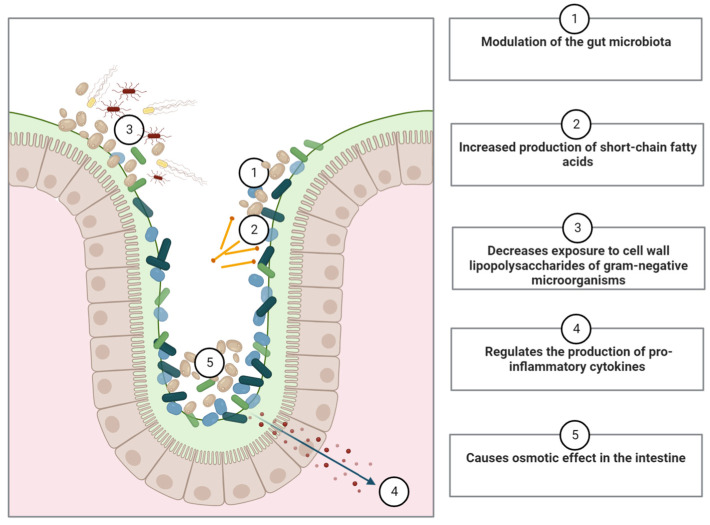
Possible mechanisms of action *of Saccharomyces boulardii* in the human body.

The results of these studies showed that ingestion of *S. boulardii* has the potential to modify the plasma and hepatic lipid profiles, as well as the amount of body fat in animal experiments [82,83,84,85]. As previously demonstrated, the administration of *S. boulardii* in patients with type 1 diabetes regulated the triglyceride secretion profile and modified the storage of triglycerides in the liver [89]. The administration of *S. boulardii* in conjunction with a hypercholesterolemic diet also resulted in a decrease in hepatic triglycerides (Table 1) [82,85]. These alterations may be directly associated with the absorption of cholesterol by the cell wall of the biomass of *S. boulardii* [85] or indirectly associated with the modulation of the intestinal microbiota [82,83] and by the regulation of pro-inflammatory cytokine production [83] (Figure 2).

However, when *S. boulardii* was given for two weeks as a treatment after consuming a high-fat diet for 8 weeks, no significant difference was found for triglyceride and total cholesterol levels [66]. Although the authors used *S. boulardii* as a treatment after diet-induced obesity, it is important to note that its use is indicated as a health promoter and not as a treatment for diseases, and, therefore, it is not regulated as a medicine by inspection agencies [90].

The cell wall of *S. boulardii* may contain β-glucan, mannoprotein, and chitin, which are known to promote cholesterol adsorption [81], resulting in the regulation of a cholesterolemic profile and a decrease in total triglyceride content that were observed in the in vivo assays (Table 1). Furthermore, the presence of β-glucans, as in the cell wall of *S. boulardii*, has been associated with beneficial health effects, such as the induction of cytokine secretion from human dendritic cells mediated by the recognition of dectin-1 [91] and colon cancer prevention [92]. In addition, β-glucans have some bioactive effects including lowering cholesterol and triglyceride levels, antioxidant potential preventing oxidative damage, and antibacterial activity [93]. For this reason, *S. boulardii*, when inactivated, has also been regarded as a postbiotic by the International Scientific Association for Probiotics and Prebiotics (ISAPP) [94], as the structures of its cell wall and the metabolites that are produced by it could also have some beneficial effects on the health of the host even under nonviable cell conditions. The administration of inactivated *S. boulardii* microorganisms may be beneficial for immunocompromised or vulnerable populations [81], as with the use of viable yeast, there is a risk of fungemia in patients [95,96].

*S. boulardii* appears to modulate the gut microbiota across phyla, genera, families, and species (Figure 2—①) in the cases of yeast administration associated with a high-fat diet in animal studies [82,83]. On the other hand, Awoyemi et al. [87] did not demonstrate significant differences in beta and alpha diversities from baseline to the end of the intervention in their phase II, multicenter, randomized, open-label, and controlled trial. These authors worked with heart failure (not exactly associated with metabolic syndrome or its risk factors) class II, III and left ventricular ejection fraction < 40% patients who were under ideal medical treatment for at least three months, including drug treatment that could influence the result demonstrated by these authors in addition to having carried out an open-label trial.

The occurrence of corpulence and obesity in individuals has been associated with a higher abundance of Bacillota (former Firmicutes) and a lower abundance of Bacteroidota (former Bacteroidetes). Additionally, obesity has been associated with an increase in the proportion of Bacillota and Bacteroidota in the intestinal microbiota and, consequently, in the feces [97]. The administration of *S. boulardii* two to four times per week appears to modulate these phyla by decreasing the abundance of Bacillota [82,83] and increasing the abundance of Bacteriodetes [83]. Furthermore, Everard, Matamoros [83] demonstrated that there may be a correlation between an increase in *Bacteroides* abundance and a decrease in fat mass, while *Prevotella* abundance was correlated with an increase in adipose tissue.

Dysbiosis is defined as an imbalance in the quantity and quality of microorganisms present in the intestinal microbiota that can occur due to genetic or environmental factors, resulting in specific diseases [98]. Dysbiosis has been widely reported as a response to an individual’s dietary habits. For example, dietary fiber intake increases colonic fermentation, resulting in increased production of short-chain fatty acids (SCFAs) that can be excreted in feces or absorbed by the intestinal epithelium to participate in a variety of physiological processes. There is still no consensus in the literature regarding the beneficial effects or not of the presence of SCFAs. However, there seems to be a correlation between the increase in the presence of these SCFAs in the feces with the diversity and composition of the intestinal microbiota, intestinal permeability and cardiometabolic results (including obesity and hypertension) [99]. Simultaneously, the presence of plasma SCFAs has been associated with positive outcomes in obesity, such as decreased metabolite translocation, induction of lipogenesis [97,100], increased triglyceride deposition by molecular pathways, and SCFAs acting as signaling molecules and activating various metabolic pathways such as the liver X receptor (LXR) pathway [97,101]. Consequently, this results in beneficial effects such as weight loss and regulation of glucose homeostasis and insulin sensitivity [101].

Administration of *S. boulardii* may directly contribute to the modulation of microbiota, resulting in decreased translocation of metabolites and cells from the intestinal lumen to the bloodstream (Figure 2—②). For example, in the cases of obesity, LPSs present in the cell membrane of Gram-negative bacteria (Figure 2—③) bind to receptor proteins (e.g., Toll-like receptor 4—TLR4) that upregulate pro-inflammatory cytokines and signaling pathways that regulate the nature, magnitude, and duration of the inflammatory response [97,102].

The LPS is an endotoxin bacterial component that is one of the most prominent TLR activators (TLR4) and can initiate the inflammatory cascade from Kupffer cells [103,104]. In turn, the increase in serum LPS, as a result of a microbiota in dysbiosis, has been related as one of the conditions that can lead to or increase the chances of the occurrence of NAFLD (nonalcoholic fatty liver disease) and fibrosis [105,106,107]. In addition, NAFLD has been recognized as the most frequent liver disease coexisting with MetS risk factors, and the onset of the disease, which is considered complex, may be linked to insulin resistance and increased adipose tissue, among other factors; therefore, it can exist as a continuum of obesity and MetS [32]. The occurrence of NAFLD is associated with oxidative stress due to excessive production of reactive oxygen species and shortage of endogenous antioxidant molecules, which induce tissue damage and promote inflammation, and is promoted by the interaction between LPS and the TLR4 system [108]. Furthermore, one must consider that the presence of LPS in the bloodstream may represent a Nox2 activation (one of the most important cellular source of oxidative stress) resulting in reactive oxygen species (ROS) production [109] which, again, is associated with the development of liver damage, inflammation, and NAFLD.

The ingestion/administration of probiotics may result in a decrease in harmful bacteria by modulating the composition of the intestinal microbiota and its metabolites and improving the barrier function, thereby decreasing the translocation and concentration of serum LPS, which delay the progression of NAFLD via the negative signaling of LPS/TLR4 [103,105,110] as well as the oxidative stress caused by the interaction between LPS and TLR4 [108].

Obesity has been shown to cause chronic inflammation in human tissue through the secretion of inflammatory proteins, and the presence of adipose tissue can further increase the secretion of these proteins by adipocytes [111]. In this sense, the presence of pro-inflammatory cytokines in tissues or in the bloodstream (such as tumor necrosis factor alpha (*TNF-α*), interleukin-1 beta (*IL-1β*) and 6 (*IL-6*), and *MCP-1*) is associated with the regulation of inflammatory responses such as proliferation and apoptosis of adipose tissue, promotion of lipolysis, inhibition of lipid synthesis, and a decrease in blood lipids [112]. *S. boulardii* has been shown to regulate liver markers (such as *CD11c*, *MCP-1*, and *IL-1B)* as well as plasma cytokines *IL-6* and *IL-4* [83] (Table 1), demonstrating its potential to decrease pro-inflammatory cytokine levels (Figure 2—④).

Administration of *S. boulardii* increased the total number of *Saccharomyces* and yeast cells [83], thereby increasing the osmotic effect in the intestine (Figure 2—⑤). The increased presence of yeast in feces can cause an osmotic effect that may be demonstrated via increased mass in the cecum [83] and increased elimination of cholesterol in feces [82].

Only one study evaluated gene expression following the administration of *S. boulardii*. Briand, Sulpice [82] reported an increase in the transcription of *ABCG5* and *ABCG8*, as has been reported by other studies on the administration of other bioactive compounds associated with a high-fat diet [113,114]. Wilund, Yu [115] stated that the increase in transcription of *ABCG5* and *ABCG8* can result in a 50% reduction in cholesterol absorption, which also seems to have occurred in the work of Briand, Sulpice [82], although not in the same proportion.

Although the assessment of the glucose profile and insulin sensitivity indices is common in studies with patients with obesity, overweight, and/or diabetes, none of the studies found for the administration of *S. boulardii* evaluated these parameters (Table 1). Rondanelli, Miraglia [116] evaluated the administration of 250 mg of *S. boulardii* (5.0 × 10^9^ CFU) together with 500 IU of superoxide dismutase (SOD) twice daily for 60 days in addition to a restrictive diet (3344 kJ/day) in patients with obesity (n = 30, BMI from 30 to 35 kg/m^2^). In addition to the decrease in body fat, BMI, and fat mass, these authors demonstrated a significant reduction in insulin levels and insulin resistance, which was reflected in the reduction in the HOMA index. This was attributed to the ingestion of *S. boulardii*, although the authors did not use a group with the probiotic alone. However, Palacios, Vitetta [117] studied the administration of a multi-strain probiotic containing *S. boulardii* for 12 weeks and found no significant differences in any glycemic parameter or insulin sensitivity index. It is possible to notice that the administration of *S. boulardii* with other agents seems to have masked its effect if a randomized group is not exclusive to the administration of this yeast.

## 4. Conclusions

Although the literature has reported some first findings regarding the administration of *Saccharomyces boulardii* in the treatment of MetS and its risk factors, research is still incipient and has limitations including the number of animals in the studies, type of biochemical evaluations, limited studies in humans, especially RCTs, among others. Therefore, for more accurate conclusions regarding the benefit of administering *S. boullardii* in this treatment, further research is needed, including a randomized controlled trial.

The works found in this review reported positive effects of *S. boullardii* administration. These effects were mainly related to the lipid profile and changes in the microbiota. Although there are some theories regarding the mechanism used by *S. boulardii* to promote health benefits, further studies are needed to elucidate these mechanisms.

In addition, to make its consumption viable, it is important to advance the development of processes that allow for its availability in a stable way through efficient carriers. Ways to do so include capsules and even food and beverages, guaranteeing its stability, viability, and dose to ensure the beneficial effects associated with this yeast consumption.

## 5. Limitations of Our Study

Although there is a beneficial potential in the ingestion of *S. boulardii,* and, therefore, it is a probiotic widely used in the daily life of health professionals, a limitation of our review was the number of studies found to be included and evaluated, as well as their heterogeneity of experimental design and results. Performing the systematic search, only five studies were found, and three were included later because they were cited in the first studies. Regardless of this, it would be impossible to carry out an evaluation compatible with those carried out in a systematic review such as a meta-analysis, since only three of these studies were carried out in human models and two of them showed secondary outcomes related to MetS. Thus, although this review is limited by bringing a narrative review of the literature with a systematic search, it is also important because it tries to explain the possible mechanisms of action of *S. boulardii* with regard to the beneficial effects related to MetS and its risk factors, and with that, it can encourage further studies to be carried out in this area.

## Figures and Tables

**Table 1:** Results of the systematic search of *Saccharomyces boulardii* administration in metabolic syndrome systems and its risk factors.

Administration (Dose/Duration)	Model	Principally Observed Results	References
Administration of *S. boulardii* CNCM I-745 (3 g/kg) by oral gavage (twice a week) for 21 or 39 days	Six-week-old hypercholesterolemic male Golden Syrian hamsters (0.3% cholesterol diet) (n = 40)	↑ 23 and 36% of total cholesterol in feces at 21 (*p* < 0.05) and 39 (*p* < 0.01) days, respectively; *HMGCoA-R* gene expression at 21 and 39 days (*p* < 0.05); and the presence of the phyla Pseudomonadota (former Proteobacteria) and Lentispharerae (*p* < 0.05). ↓ plasma total cholesterol (*p* < 0.001); 31% HDL-c in 21 days; 25% hepatic triglycerides (*p* < 0.05) in 39 days; 43% of the total mass of bile excreted in feces in 21 days; *ABCG5* gene expression at 21 and 39 days (*p* < 0.05); *ABCG8* gene expression in 39 days (*p* < 0.05); and the presence of phyla Bacillota, Tenericutes, and TM7 (*p* < 0.05).	Briand et al. [82]
Daily administration of *S. boulardii* Biocodex (120 mg) by oral gavage for 4 weeks	Six-week-old mice with leptin resistant obesity and type 2 diabetes (db/db) (n = 30)	↑ mass of cecum and cecum tissue; 160 times total Saccharomyces and 40 times total yeast cells; 37% phylum Bacteroidota; 6 times the Bacteroidaceae family; and 40 times the genus *Bacteroides*. ↓ 15% mass gain; fat mass; liver mass; adipose index; visceral, epididymal, and subcutaneous fat; total fat content in the liver; markers of hepatic macrophage infiltration (50% for *CD11c*, 40% for *MCP-1,* and 37% for *IL-1B*); plasma concentrations of cytokines (*IL-6* and *IL-4* twice); 57, 55, and 30% the phyla Tenericutes, Proteobacteria, and Bacillota; 8 times the Porphyromonadaceae family; and genera *Anaeroplasma* (92%), *Anaerotruncus* (47%), *Dorea* (77%), *Odoribacter* (82%), *Oscillospira* (38%), *Parabacteroides* (91%), *Prevotella* (76%), and *Ruminococcus* (44%).	Everard et al. [83]
Oral administration of *S. boulardii* at 12 × 10^10^ CFU/kg (3 g/kg) twice a day in two experimental designs, being (1) a preventive protocol for 14 days from the beginning of the diet and (2) a curative protocol after a 14-day curative protocol with *S. boulardii* starting after 14 days of cholesterolemic diet	Male Golden Syrian hamsters (weighed 80–90 g, 0.1% cholesterol)	↓ (1) 14% increased cholesterolemia and 26% increased liver cholesterol; and (2) 12% increase in cholesterolemia and 39% increase in total plasma triglycerides.	Girard et al. [84]
*S. boulardii* (Lupin Laboratories, India) was administered at a concentration of 10^8^ CFU/g along with the diet (0.5% cholesterol, 30% dalda, 10% refined oil and 50% wheat flour) with high lipid concentration for 21 (1) and 42 (2) days	Adult male Wistar rats (mean body weight 150 g, n = 12)	↓ (1) total triglycerides (108.12 to 85.63 mg/dL) and total cholesterol (98.88 to 74.70 mg/dL); (2) total triglycerides (260.20 to 108.73 mg/dL), total cholesterol (173.70 to 65.63 mg/dL), LDL-c (110.37 to 27.55 mg/dL), atherogenic index (9.67 to 3.05), LDL/HDL (6.82 to 1.64), and body mass (314.16 to 300.83 g).	Saikia et al. [85]
*Saccharomyces boulardii* CNCM I-745 (Yomogi^®^) (Ardeypharm GmbH, Herdecke, Germany) was administered at a concentration of 10^9^ yeast cell/rat daily by oral gavage for 2 weeks (14 d)	Male Sprague-Dawley rats (8-week-aged and body weight to 150–180) assigned a high-fat diet for 8 weeks (body weight to 350–400 g)	After two weeks of treatment with *S. boulardii*, there was no significant change in triglyceride and total cholesterol levels, as well as body weight of the rats.	Nayebhashemi et al. [66]
Two capsules of 250 mg of the *S. boulardii (CNMI-745)* twice a day for 3-month oral daily therapy	Heart failure patients (n = 46, phase II, multicenter, randomized, open-label, controlled trial)	↑ N-terminal-pro-B-type-natriuretic peptide level. No significant difference these was demonstrate for C-reactive protein, trimethyl-mine N-oxide (TMAO), and global microbiota composition (beta diversity) and bacterial richness (a diversity) from baseline to the end of the intervention group.	Awoyemi et al. [87]
*S. boulardii* (1000 mg) per day for 3-month oral daily therapy	Heart failure patients (n = 7, randomized, double-blind, placebo-controlled pilot trial)	↓ total cholesterol (150.8 to 143.2 mg/dL); uric acid (6.15 to 5.1 mg/dL); creatinine (1.12 to 0.9 mg/dL); high-sensitivity C reactive protein (0.65 to 0.22 mg/dL); and left atrial diameter (4.5 to 4.2 cm).	Costanza et al. [88]
*S. boulardii* CNCM I-1079 (5.6 × 10^10^ CFU/g) per capsule twice daily for a period of 8 weeks	Participants (n = 11) hypercholesterolemic (200–275 mg/dL and BMI of 20–45 kg/m^2^) aged 21–69 years	↓ remnant lipoprotein particles (*p* = 0.03).Nonsignificant results in the variables measured related to the MetS.	Ryan et al. [86]

↑: increased and ↓: decreased.

## Data Availability

Not applicable.
